# High expression of TRMT112 is associated with the development of oral squamous cell carcinoma

**DOI:** 10.1016/j.jobcr.2025.12.014

**Published:** 2026-01-06

**Authors:** Anitha Pandi, Premkumar Rajendhiran, Vijayashree Priyadharsini Jayaseelan, Paramasivam Arumugam

**Affiliations:** a Clinical Genetics Lab, Saveetha Dental College and Hospital, Saveetha Institute of Medical and Technical Sciences (SIMATS), Saveetha University, Chennai, India; b Molecular Biology Lab, Saveetha Dental College and Hospital, Saveetha Institute of Medical and Technical Sciences (SIMATS), Saveetha University, Chennai, India

**Keywords:** OSCC, TRMT112, Mortality, Prognosis, Therapeutic target

## Abstract

**Background:**

TRMT112 is a member of the transfer RNA (tRNA) methyltransferase family, and its dysregulation in humans is involved in carcinogenesis. This study aimed to investigate the expression and clinical significance of TRMT112 in patients with oral squamous cell carcinoma (OSCC).

**Materials and methods:**

Quantitative real-time PCR (qPCR) and Western blot were used to analyze TRMT112 expression in paired tumor and non-tumor tissues of OSCC. Furthermore, we analyzed TRMT112 expression for clinicopathological features, prognosis, immune infiltration, and immunotherapy response using the TCGA-HNSCC datasets, which primarily include OSCC data through UALCAN, Human Protein Atlas, Kaplan-Meier plots, and TIMER2.0. The oncogenic role and mechanism of TRMT112 were analyzed using a functional enrichment approach.

**Results:**

TRMT112 expression was significantly upregulated in OSCC tissues compared to non-tumor tissues. The upregulated expression of TRMT112 was associated with advanced tumor stages, metastasis, lower immune infiltrating levels, immunotherapy resistance, and worse prognosis. Protein network and functional pathway enrichment analysis revealed that TRMT112 interacts with well-known oncoproteins that play a critical role in oral cancer progression.

**Conclusions:**

Overall, our novel findings revealed that TRMT112 is associated with the oncogenic process of OSCC, which suggests that TRMT112 could serve as a potential prognostic and therapeutic candidate.

## 1 Introduction

Oral squamous cell carcinoma (OSCC) is the most common cancer found in the oral cavity and continues to be a significant factor in cancer-related illness and death globally. Its poor survival outcomes mainly result from delayed diagnosis and limited treatment options 1, 2, 3. Hence, unraveling the molecular mechanisms that drive OSCC development is essential for designing effective diagnostic tools and therapeutic strategies. Both genetic mutations and epigenetic modifications have been implicated in OSCC pathogenesis. More recently, RNA-based epigenetic regulation, often referred to as epitranscriptomics, has emerged as a critical player in tumor initiation and progression, acting as a novel insight into the biology of this malignancy^4^, ^5^, ^6^.

N6-methyladenosine (m6A) is the most common internal RNA modification found in eukaryotic cells and has recently attracted significant interest due to its biological importance^7^ .^8^ This active and reversible alteration affects RNA outcomes by controlling splicing, stability, nuclear export, and translation, processes facilitated by three categories of proteins: methyltransferases (“writers”), demethylases (“erasers”), and RNA-binding proteins (“readers”) 9, 10, 11, 12. Accumulating evidence indicates that aberrant m6A regulation contributes to cancer onset and progression through diverse molecular mechanisms. Importantly, the study of m6A dynamics has opened new avenues for early cancer detection and targeted therapeutic interventions 13, 14, 15.

Transfer RNA methyltransferase subunit 11-2 (TRMT112) functions as an essential cofactor in multiple RNA methylation processes, particularly those involving transfer RNA (tRNA). Methylation of tRNA is vital for maintaining translational fidelity and efficiency, ensuring the correct synthesis of proteins.^16^^,^^17^ TRMT112 participates in this process by facilitating the addition of methyl groups to specific nucleotide positions within tRNA. Beyond tRNA regulation, TRMT112 forms a complex with METTL5, an m6A-associated methyltransferase, which catalyzes the modification of adenosine at position 1832 of human 18S rRNA.^18^^,^^19^ METTL5, a newly discovered regulator, has been associated with cancer biology due to its capability to influence gene expression in an m6A-dependent manner. Despite this knowledge, the exact role of TRMT112 in OSCC tumorigenesis and its underlying molecular mechanisms still remain unclear.^20^^,^^21^

The current research aimed to outline the expression profile and cancer-causing potential of TRMT112 in OSCC. To this end, we evaluated its expression in tumor samples along with matched adjacent non-tumor tissues, using both experimental validation and computational analyses. Furthermore, a comprehensive analysis was conducted on the association between TRMT112 expression and clinicopathological characteristics, patient outcomes, and the infiltration of immune cells. Functional interaction networks and pathway analyses were also performed to elucidate the mechanisms through which TRMT112 may contribute to OSCC progression. Collectively, our findings indicate that TRMT112 has a potential oncogenic role with significant prognostic and therapeutic implications in OSCC.

## 2 Materials and methods

### 2.1 Tissue specimens

A total of 38 matched specimens of OSCC and adjacent non-tumor tissues were collected during surgeries at the Department of Oral and Maxillofacial Surgery, Saveetha Dental College and Hospitals in Chennai. The criteria for inclusion required primary, histologically confirmed OSCC with corresponding adjacent normal tissue, while the exclusion criteria involved previous chemotherapy or radiotherapy and recurrent lesions with any other primary tumor and systemic disorders. Samples were immediately preserved at −80 °C until subsequent analyses. Histopathological evaluation was used to confirm both malignant lesions and matched non-tumor tissues. The Institutional Ethics Committee approved the study (IEC NO: SDC/FAC-12/19/003) which was carried out following the guidelines of the Declaration of Helsinki. Before sample collection, written informed consent was obtained from all participants. Baseline characteristics and clinicopathological information of patients are listed in Supplementary Table 1.

### 2.2 Cell culture

CAL27, SCC25, and SCC15 OSCC cell lines were obtained from the American Type Culture Collection (ATCC, USA) and confirmed to be negative for mycoplasma. For comparison purposes, normal human oral keratinocytes (NHOK/OKF6) were included as detailed in previous research.^22^ OSCC cells were cultured in Dulbecco's Modified Eagle Medium (DMEM) enriched with 10 % fetal bovine serum (FBS) and 1 % penicillin-streptomycin. NHOK cells were cultured in a medium specific to keratinocytes. All cultures were maintained at 37 °C in a humidified atmosphere with 5 % CO_2_

### 2.3 mRNA expression analysis

TRIzol reagent (Invitrogen, Waltham, MA, USA) was employed to isolate RNA from OSCC tissues and cell lines according to the manufacturer's protocol. Reverse transcriptase-dependent cDNA synthesis was performed using an RT Kit (Takara Bio Inc., Tokyo, Japan, Cat. #6110A). For mRNA expression analysis, the qPCR kit (Bio-Rad, USA, Cat. #1725271) was utilized, along with the CFX Opus Real-Time PCR System (Bio-Rad, USA). The primers used for TRMT112 in this study are forward 5′-GGCCGATAACTTGCGTCTGA-3′, Reverse 5′-GGGTGCCCTCTATCACTTCC-3’. The mRNA was quantified using the expression of glyceraldehydes 3-phosphate dehydrogenase (GAPDH) as an internal control, and the primer sequences for GAPDH are Forward- 5′-TCCAAAATCAAGTGGGGCGA-3′ and Reverse 5′-TGATGACCCTTTTGGCTCCC-3’. The qPCR was performed, and the relative mRNA expression was determined using the 2^−ΔΔCt^ method. Additionally, the Cancer Genome Atlas-(TCGA-OSCC/HNSCC) dataset was used to study TRMT112 expression and its association with clinical and pathological characterisation, prognosis, and infiltration of immune cells *via* UALCAN (updated version 2022) (http://ualcan.path.uab.edu). database,^23^ the Kaplan-Meier plot (https://kmplot.com/),^24^ Oncolnc (http://www.oncolnc.org/),^25^ TIMER 2.0 (http://timer.cistrome.org),^26^ and TNMplot (https://tnmplot.com/analysis/) respectively. The TCGA-HNSCC dataset was also used in this study to analyze TRMT112 expression and its clinicopathological characteristics because it contains a large tumor sample size (n = 520), among which the cases of OSCC constitute the largest group (>65 %). The HPV status (positive/negative) was determined from the clinical annotations provided by TCGA-HNSCC. The clinical characteristics of patients with HNSCC from the TCGA-HNSCC dataset are listed in Supplementary Table 2.

### 2.4 Protein expression analysis

The protein was extracted using RIPA lysis buffer, and its concentration was measured with the bicinchoninic acid (BCA) method. Equal amounts of protein were loaded onto an 8 % or 10 % SDS-PAGE gel for separation and subsequently transferred to a PVDF membrane (Millipore, MA, USA). The membranes were then blocked with 5 % non-fat milk and incubated with primary antibodies of TRMT112 (Abclonal, Cat. A14310; dilution 1:1000) and GAPDH (Santa Cruz Biotechnology, Cat. sc-47724; dilution 1:1000), followed by incubation with a secondary antibody (Santa Cruz Biotechnology, Cat. sc-2357; dilution 1:2000 and Abclonal, Cat. AS002; dilution 1:2000). Detection of the signals was carried out using an enhanced chemiluminescence (ECL) detecting method (ChemiDoc XRS + System, BioRad). The internal reference protein for this experiment was GAPDH. In addition, the TRMT112 protein expression level was analyzed using UALCAN and Human Protein Atlas databases (https://www.proteinatlas.org/).^27^

### 2.5 Functional analysis of TRMT112 using *in silico* tools

Protein–protein interaction networks were explored using the STRING v12.0 (https://string-db.org) database. The functional enrichment related to TRMT112, encompassing molecular functions, biological processes, and cellular components, was evaluated using Metascape v3.5. (https://metascape.org). Correlation analyses between gene expression profiles were performed using the cBioPortal platform (https://www.cbioportal.org/).

### 2.6 Statistical analysis

Statistical evaluations were performed using GraphPad Prism 10.1.2 (GraphPad Software). Statistical significance was determined at a *P* value of less than 0.05. To compare groups, analysis of variance (ANOVA), Student's t-test, and Pearson's correlation were applied. The Kaplan-Meier method, along with the log-rank test, was used to assess patient survival. Results are shown as the mean deviation from two or three independent experiments.

## 3 Results

### 3.1 TRMT112 was highly expressed and associated with poor prognosis in OSCC

**Fig. 1:**
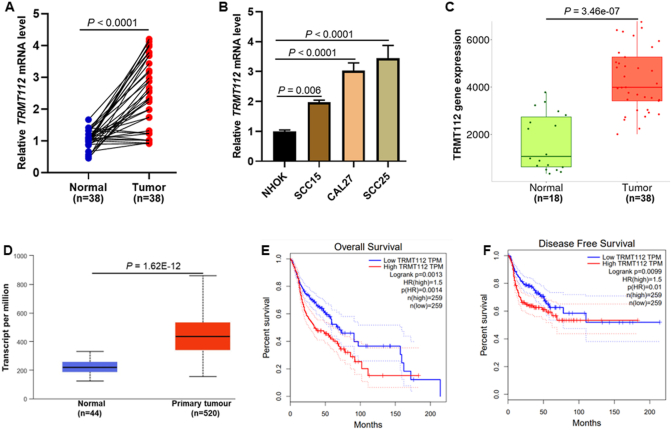
TRMT112 was highly expressed in OSCC and associated with poor prognosis. (A) qPCR analysis showed that *TRMT1*12 mRNA was significantly increased in OSCC tissues compared to matched adjacent non-tumor tissues. (B) qPCR analysis revealed that *TRMT1*12 mRNA was highly expressed in OSCC cell lines when compared to normal oral keratinocyte cell line. (C) *TRMT1*12 mRNA was significantly overexpressed in the OSCC-TCGA dataset. (D) *TRMT1*12 mRNA was significantly overexpressed in the HNSCC-TCGA dataset. (E, F) Kaplan-Meier and log-rank testing showed the prognosis of the patient cohort (overall and disease-free survival).

To assess the relationship between TRMT112 expression and OSCC, we initially evaluated the mRNA levels of *TRMT112* in 38 OSCC samples, along with their corresponding non-tumor tissues using qPCR. Our findings revealed that *TRMT1*12 mRNA expression was significantly higher in OSCC tumor tissue compared to matched adjacent non-tumor samples (*P* < 0.0001; Fig. 1A). Consistent findings were observed in OSCC-derived cell lines (SCC25, CAL27, and SCC15), which exhibited higher TRMT112 expression relative to normal oral keratinocytes (*P* < 0.001; Fig. 1B). To validate these results in a larger cohort, we examined RNA-seq datasets from TCGA-OSCC (*P* = 3.46e-07; Fig. 1C). and TCGA-HNSCC (*P* = 1.62e-12; Fig. 1D), which predominantly represent OSCC cases. Both datasets confirmed significantly higher *TRMT1*12 mRNA levels in tumor tissues compared to non-tumor tissues. Survival analyses that utilized Kaplan-Meier curves and Cox regression consistently indicated that *TRMT112* overexpression correlates with poor overall survival in HNSCC. In KM analyses, higher levels of TRMT112 expression were significantly associated with decreased overall survival (*P* = 0.001; Fig. 1E) and disease-free survival (*P* = 0.009; Fig. 1F) among patients, highlighting its potential role as a prognostic marker. This unfavorable prognostic pattern was consistently observed in all key clinical subgroups. This suggests that the adverse effect of TRMT112 is largely independent of demographic or histological factors. In line with this, the OncoLnc univariate Cox model showed a positive hazard coefficient (*β* = 0.149) and statistical significance (*p* = 0.029), thus confirming that HNSCC patients with higher TRMT112 expression have a higher risk of death.

**Fig. 2:**
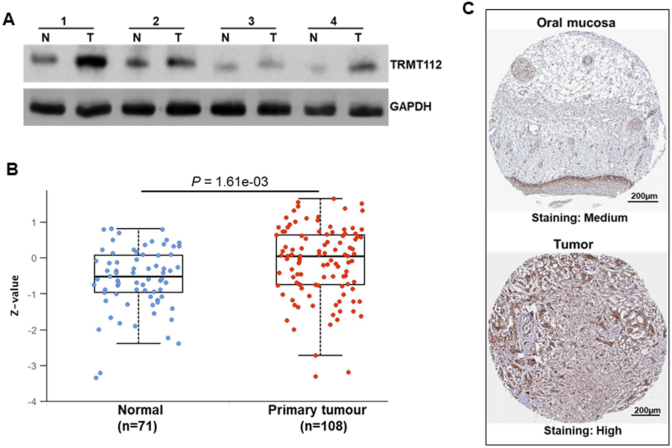
Protein expression of TRMT112 in OSCC tissues. (A) Western blot analysis showed that TRMT112 protein expression significantly increased in OSCC compared with adjacent non-tumor tissues. (B) HNSCC-TCGA dataset revealed that the expression of TRMT112 was up-regulated in HNSCC tissues compared to normal tissues. (C) Immunohistochemistry analysis also indicated that TRMT112 was highly expressed in tumor tissues when compared to normal tissues.

Western blot analysis additionally validated that the levels of TRMT112 protein were significantly elevated in OSCC tissues compared to nearby non-tumor tissues (Fig. 2A). Supporting evidence analyses using UALCAN (*P* = 1.61e-03; Fig. 2B) and the Human Protein Atlas (Fig. 2C), based on TCGA-HNSCC datasets, revealed a similar trend, with protein expression consistently elevated in malignant tissues compared to normal samples, suggesting that TRMT112 is significantly overexpressed in OSCC and is linked to unfavorable clinical outcomes.

### 3.2 Correlation between increased expression of TRMT112 and clinical parameters

**Fig. 3:**
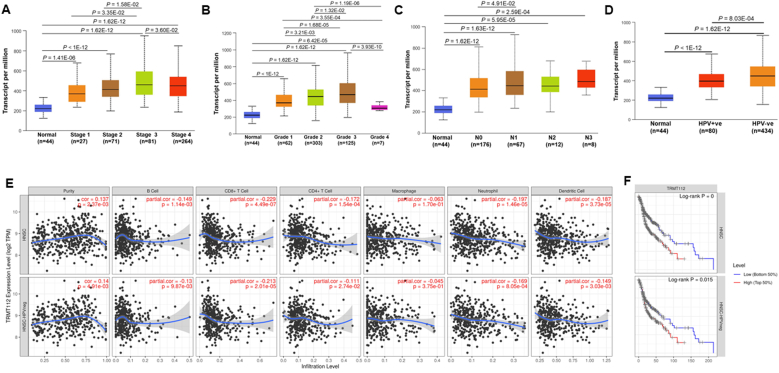
*TRMT112* mRNA expression significantly correlated with the clinicopathological features of HNSCC such as tumor stage (A), Tumor grade (B), nodal metastasis (C), HPV status (D), immune cells infiltration (E), and poor prognostic value of infiltrating immune cells, especially HPV-negative HNSCC patients (F).

Analysis of the UALCAN database showed that increased expression of TRMT112 was strongly associated with the higher histological grades, advanced tumor stages, nodal metastasis, and HPV status in patients with HNSCC (*P* < 0.05; Fig. 3A–D). Recognizing the pivotal role of immune cell infiltration in influencing the tumor microenvironment, additionally evaluated the correlation between TRMT112 expression and immune infiltration utilizing TIMER 2.0. The analysis revealed a significant correlation between the expression of TRMT112 and various immune cell types. (*P* < 0.05; Fig. 3E). Notably, TRMT112 levels showed an inverse correlation with CD4^+^ T lymphocytes, macrophages, and neutrophils. Moreover, high TRMT112 expression was particularly linked to poorer immune-related prognosis in HPV-negative HNSCC patients, suggesting its potential role in modulating tumor–immune interactions (*P* = 0.01; Fig. 3F). These results indicated that the elevated levels of TRMT112 expression were significantly linked to the clinical and pathological characteristics of patients with HNSCC.

**Fig. 4:**
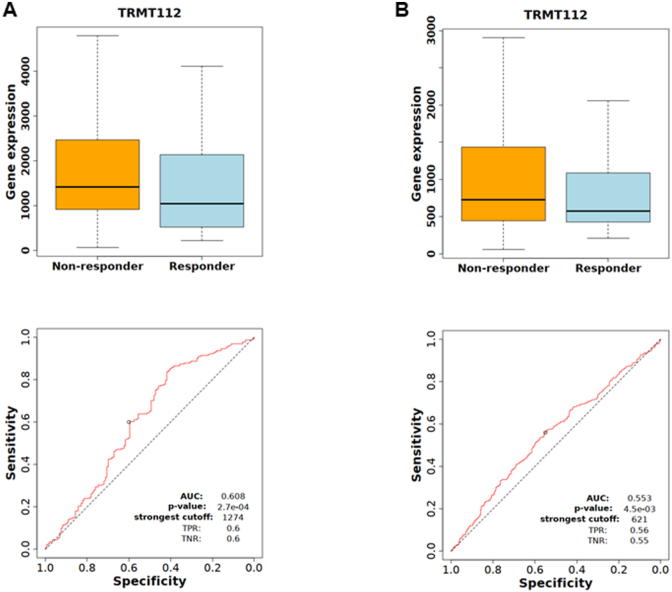
TRMT112 expression and immunotherapy resistance. High expression of TRMT112 is associated with resistance to anti-PD-1 (A) and anti-CTLA-4 (B) treatments in patients with metastatic HNSCC.

In addition, elevated TRMT112 expression was associated with reduced responsiveness to immune checkpoint blockade, specifically anti-PD-1 (*P* = 2.7e-04; Fig. 4A) and anti-CTLA-4 (*P* = 4.5e-03; Fig. 4B) therapies in patients with metastatic HNSCC. Statistical analysis revealed that higher TRMT112 levels corresponded with diminished treatment efficacy, as reflected by AUC values of 0.608 for anti-PD-1 and 0.553 for anti-CTLA-4, both reaching statistical significance. Optimal threshold values for distinguishing responders from non-responders were approximately 1274 and 621 expression units, with moderate sensitivity and specificity (∼0.6). These observations suggest that TRMT112 upregulation may act as a predictive marker for resistance to immunotherapy in advanced HNSCC patients.

### 3.3 Functional and pathway enrichment analysis of TRMT112

**Fig. 5:**
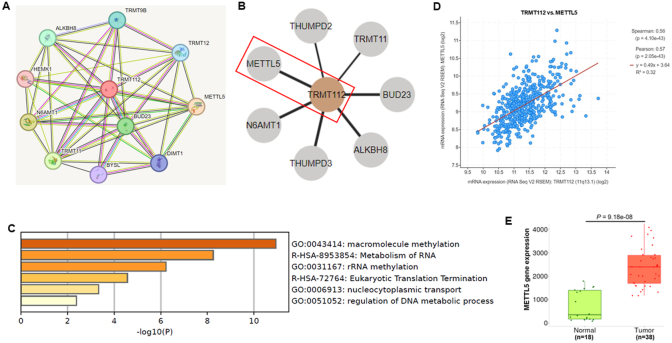
Networks, pathways, and diseases associated with TRMT112. (A, B) TRMT112 protein network. (C) Functional enrichment analysis of the TRMT112 protein interactions associated with cancer-linked pathways. (D) TRMT112 expression positively correlated with METTL5 expression. (E) High expression of METTL5 in OSCC patients (TCGA-OSCC dataset).

The protein interaction network of TRMT112 was examined utilizing the STRING and Human Protein-Atlas databases, which provide information regarding protein-protein interactions. Interestingly, several well-known oncogenic proteins, including METTL5, ALKBH8, TRMT11, THUMPD2, THUMPD3, N6MT1, BUD23 are mainly interacted with TRMT112 (Fig. 5A and B). Additionally, we analyzed functional pathway enrichment, which indicated that TRMT112 and its associated network are linked to RNA metabolism, rRNA methylation, the termination of eukaryotic translation, nucleocytoplasmic transport, the regulation of DNA metabolic processes, and the methylation of macromolecules (Fig. 5C). These are established pathways that are crucial for the proliferation, growth, and survival of cancer cells. Therefore, our findings suggest that targeting TRMT112 or its associated pathway represents a promising approach for treating OSCC.

METTL5 is a methyltransferase that catalyzes the m6A modification of 18S rRNA, a process known to enhance the translational efficiency of several oncogenic proteins and thereby promote tumor development. Recent evidence has shown that TRMT112 serves as the key interacting partner of METTL5, playing a crucial role in maintaining its metabolic stability. The information from the TCGA dataset showed that TRMT112 was positively associated with METTL5 in patients with HNSCC (*P* = 4.10e-43; Fig. 5D). Additionally, the TCGA-OSCC dataset revealed a significantly higher expression level of METTL5 in OSCC tissues when compared to normal tissues (*P* = 9.18e-08; Fig. 5E). The pathways that were found to be enriched indicate that TRMT112 could be instrumental in the development and progression of cancer, potentially collaborating with the METTL5 oncogene. However, further molecular studies are needed to understand the oncogenic role of the TRMT112-METTL5 axis in the pathogenesis of OSCC.

## 4 Discussion

TRMT112 is a compact, well-conserved eukaryotic protein that functions as a crucial regulatory partner for various methyltransferases. Its functional role was first described in *Saccharomyces cerevisiae*, and subsequent work in mammalian systems confirmed its broad participation in RNA and protein methylation pathways.^28^ In mammalian cells, TRMT112 is localized in both the nucleus and cytoplasm, forming specific complexes with distinct methyltransferases. It associates in the nucleus with WBSCR22 and THUMPD2, interacts with N6AMT1 and METTL5 in both compartments, and forms predominant complexes in the cytoplasm with THUMPD3, ALKBH8, and TRMT11. These compartment-specific interactions highlight TRMT112's function as a versatile scaffold protein, promoting the stability and catalytic efficiency of its binding partners.^16^^,^^29^^,^^30^

Our study is the first to demonstrate the oncogenic contribution of TRMT112 in the development of OSCC. We observed significantly increased TRMT112 expression in both primary OSCC tissues and cell lines, which correlated with unfavorable patient outcomes. Analysis of TCGA datasets further confirmed that TRMT112 is considerably overexpressed across multiple malignancies, including breast, bladder, colon, esophageal, head and neck, renal clear cell, hepatocellular, and lung squamous cell carcinomas. Moreover, our investigation of TRMT112 revealed strong links to advanced cancer stages, higher tumor grades, nodal metastasis, TP53 mutations, HPV status and poorer outcomes in HNSCC, indicating its role in cancer progression. Earlier studies have suggested that the dysregulation of TRMT112 may play a role in the advancement of cancer.^31^

As a cofactor, TRMT112 plays a stabilizing role in regulating WBSCR22 by maintaining its protein abundance, ensuring proper nuclear localization, and preventing degradation through the proteasome pathway. This conserved mechanism is essential for ribosome biogenesis. In the context of OSCC, abnormal TRMT112 expression may disrupt WBSCR22 stability, thereby altering ribosome assembly and driving uncontrolled protein synthesis, ultimately contributing to malignant transformation. Accumulating evidence links TRMT112-mediated methyltransferase activity to critical cellular processes, including ribosome production, protein translation, and proliferation. These functions are increasingly associated with cancer development, supporting the notion that TRMT112 not only contributes to tumorigenesis but may also serve as a prognostic biomarker across multiple cancer types.^32^;^33^

In this study, we examined TRMT112 expression and its functional roles in OSCC. TRMT112 expression in OSCC tissues was significantly higher than in adjacent non-tumoral tissues. Interestingly, the results from this study were consistent with the analysis conducted in the TCGA dataset, which again confirmed higher TRMT112 expression in OSCC tissues compared to normal tissues. High TRMT112 expression levels showed a significant correlation with clinicopathological features, prognosis, tumor-infiltrating immune cells, and resistance to immunotherapy. The analysis of immunotherapy responses is still in its early stages; the results are categorized as exploratory. The observed correlations suggest a potential predictive significance; however, the modest AUC values indicate that the model's ability to differentiate remains quite limited. As a result, these findings should be viewed as generating hypotheses rather than confirming them, and future validation through larger characterization cohorts is necessary for clinical application to be established.

TRMT112 has been found by recent study to be a highly conserved protein that acts as both a cofactor and activator for different methyltransferases involved in the methylation of rRNA, tRNA, and proteins. Our network analysis confirmed that TRMT112 directly interacts with METTL5, and their expression levels are positively correlated in patients with OSCC. Functional enrichment further demonstrated that the TRMT112–METTL5 network is associated with RNA metabolic processes, ribosomal methylation, translation termination, nucleocytoplasmic transport, and DNA metabolic regulation, biological pathways frequently implicated in oncogenesis and tumor progression. Corroborating the above, the recent literature has suggested that an elevated expression of METTL5 is associated with aggressive clinical outcomes in various cancer types.^20^^,^^34^ Analysis of TCGA datasets similarly demonstrated METTL5 upregulation in diverse malignancies, including OSCC, where it is linked to unfavorable prognosis. This is the first report to provide clinical and *in silico* functional evidence that TRMT112 is required for the development and progression of OSCC.

Our previous studies have established that METTL5 is significantly overexpressed in oral squamous cell carcinoma (OSCC), correlating with advanced tumor stage, reduced survival rates, and alterations in immune response.^35^ The present investigation into the heightened expression of TRMT112, previously demonstrated by van Tran et al. (2019) as the cofactor essential for the stabilization of METTL5 for the methylation of 18S rRNA at m^6^A1832, reinforces the existence of a TRMT112–METTL5 oncogenic axis in OSCC.^19^ Biochemical analyses confirm that METTL5 requires TRMT112 as a crucial stabilizing cofactor to assemble an active 18S-rRNA m^6^A1832 methyltransferase complex. The work of Sepich-Poore et al. (2022) illustrated the co-immunoprecipitation between METTL5 and TRMT112, their colocalization within the nucleolus, the TRMT112-mediated stability of METTL5, and the loss of 18S-rRNA m^6^A1832 upon depletion of METTL5, while structural investigations underscore TRMT112's role as a vital activator of METTL family enzymes.^18^^,^^36^ Given this, our finding of elevated TRMT112 expression in OSCC highlights the biological significance of a TRMT112–METTL5 oncogenic pathway, which may be involved in disrupted rRNA methylation and the ensuing oncogenic activities. The connection established in this research will be confirmed through continuing mechanistic studies that seek to elucidate the causative nature of this pathway and showcase its translational relevance. Additionally, the recent emergence of small RNA-based therapies, including miRNA mimics, long non-coding RNA (lncRNA) sponges, antisense oligonucleotides, and RNA-guided delivery mechanisms, underscores the potential of these strategies for cancer treatment by targeting dysregulated RNA-modifying enzymes^37^, ^38^, ^39^. These innovative methods may provide a means to influence TRMT112 or its related methylation processes, positioning this pathway as a prospective target for precision RNA-based therapy in OSCC. Future research will investigate the role of TRMT112 and METTL5 in translation and clinical contexts, focusing on their impact on therapy responses and patient outcomes. These studies may employ small RNA-based strategies to target TRMT112, METTL5, or associated rRNA methylation, and assess their therapeutic significance in OSCC.

A major advantage of this research is the consistency observed between results from experiments on matched OSCC tissue samples and findings from the independent TCGA dataset. This validation through different methods not only strengthens the robustness of the observed TRMT112 upregulation but also underscores its broad significance. Additionally, the combination of transcriptomic, proteomic, and bioinformatic analyses, along with a rigorous paired design and statistical validation, has significantly increased the credibility of our findings. It is important to recognize the limitations of our study, even though it offers valuable insights into the role of TRMT112 in OSCC. These limitations include the small sample size, the focus on a single center, and the absence of functional experiments. Future research should involve larger, multi-center cohorts and mechanistic studies to clarify the biological and clinical significance of TRMT112-METTL5 interactions in OSCC.

## 5 Conclusion

In conclusion, our study provides strong evidence that TRMT112 plays a role in the malignant progression of OSCC. These findings indicate that TRMT112 could serve as both a prognostic marker and a potential therapeutic target, opening new avenues for improving patient management with this cancer.

## Patient consent

Not Applicable.

## Authors contribution

A.P. curated the data from the databases, performed the methodology, and wrote the original draft. P.R. performed the methodology and formal analysis. V.P.J. collected the data and performed formal analysis. P.A. contributed to the conception and design of the study, analyzed and interpreted the data, and supervised. All authors reviewed the results and approved the final version of the manuscript.

## Ethical clearance

Ethical clearance for the study was obtained.

Institutional Human Ethical Committee (IHEC) with reference number (IEC NO: SDC/FAC-12/19/003)

## Declaration of generative AI in scientific writing

During the preparation of this work, the author(s) used an AI tool to improve English readability. After using this tool, the author(s) reviewed and edited the content as necessary and accept responsibility for the publication's content.

## Funding information

This work was supported by Saveetha Dental College and Hospitals, SIMATS, Chennai-600077.

## Declaration of competing interest

The authors declare that they have no known competing financial interests or personal relationships that could have appeared to influence the work reported in this paper.

## Data Availability

Data generated or analyzed during this study are downloaded from TCGA datasets.
