# Supplementary material for: High expression of TRMT112 is associated with the development of oral squamous cell carcinoma
Source: J Oral Biol Craniofac Res. 2026 Jan 6;16(1):273–8. doi: 10.1016/j.jobcr.2025.12.014 (PMC12811455; doi:10.1016/j.jobcr.2025.12.014)
Supplement: Multimedia component 1 [file mmc1.docx]

**Supplementary Table 1: Clinical features of patients with oral squamous cell carcinoma**

| **S.No.** | **Variable** | **Category** | **No. of patients (%)** |
| --- | --- | --- | --- |
| 1 | Gender | Male | 31 (81.6) |
|  |  | Female | 7 (18.4) |
| 2 | Age | < 50 years | 15 (39.5) |
|  |  | > 51 years | 23 (60.5) |
| 3 | Grade | Well differentiated | 22 (57.9) |
|  |  | Moderately differentiated | 14 (36.8) |
|  |  | Poorly differentiated | 2 (5.3) |
| 4 | Site | Buccal | 12 (31.6) |
|  |  | Tongue | 8 (21) |
|  |  | Other (RMT, GBS, Maxilla, Mandible) | 18 (47.4) |
| 5 | Stage | I | 5 (13.2) |
|  |  | II | 8 (21) |
|  |  | III | 7 (18.4) |
|  |  | IV | 18 (47.4) |
| 6 | Laterality | Left | 14 (36.8) |
|  |  | Right | 24 (63.2) |
| 7 | Lymph node metastasis | Yes | 15 (39.5) |
|  |  | No | 23 (60.5) |

RMT- Retromolar trigone, GBS – gingivobuccal sulcus.
